# Encoding Magnetic States in Monopole‐Like Configurations Using Superconducting Dots

**DOI:** 10.1002/advs.201600207

**Published:** 2016-09-14

**Authors:** Anna Palau, Sergio Valencia, Nuria Del‐Valle, Carles Navau, Matteo Cialone, Ashima Arora, Florian Kronast, D. Alan Tennant, Xavier Obradors, Alvaro Sanchez, Teresa Puig

**Affiliations:** ^1^ Institut de Ciència de Materials de BarcelonaICMAB‐CSICCampus de la UAB08193BellaterraSpain; ^2^ Helmholtz‐Zentrum Berlin für Materialien und EnergieAlbert‐Einstein‐Strasse 15D‐12489BerlinGermany; ^3^ Grup d'ElectromagnetismeDepartament de FisicaUniversitat Autonoma de Barcelona08193Bellaterra, BarcelonaSpain; ^4^ Neutron Sciences DirectorateOak Ridge National Laboratory1 Bethel Valley RdOak RidgeTN37831USA

**Keywords:** micromagnetic simulations, monopolar fields, spin textures, superconductor‐ferromagnetic hybrids, XMCD PEEM images

## Abstract

**A large manifold of nontrivial spin textures**, **including the stabilization of monopole‐like fields**, are generated by using a completely new and versatile approach based on the combination of superconductivity and magnetism. Robust, stable, and easily controllable complex spin structures are encoded, modified, and annihilated in a continuous magnetic thin film by defining a variety of magnetic states in superconducting dots.

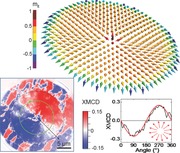

The emergence of nontrivial magnetic states such as vortices, skyrmions, and monopoles has extended the boundaries of magnetism and at the same time opened up novel technological possibilities for controlling and manipulating magnetic and electronic states.^1^, ^2^, ^3^, ^4^ Especially attractive are artificial monopole‐like defects because of their similarity to magnetic charges. They can be generated in spin ice materials or in the form of head‐to‐head domain walls in magnetic nanowires,^5^, ^6^, ^7^, ^8^, ^9^, ^10^, ^11^, ^12^ however controllable approaches to imprint and manipulate such spin structures are needed. Here we present a novel strategy for robustly encoding nontrivial magnetic states in artificial nanoscale hybrid systems, formed by a soft ferromagnetic (FM) thin film interacting with hard superconducting thin dots. By taking advantage of the control of different remanent states that can be achieved in the superconducting dots, a large manifold of magnetic patterns can be imprinted, stabilized, modified, and annihilate in the ferromagnetic layer. The obtained configurations include head‐to‐head magnetic structures yielding a monopolar field with controlled polarity. Micromagnetic simulations confirm that the magnetic structures appearing in the ferromagnet are arising and governed from the stray fields created by superconducting dots. Hence, complex magnetic structures can be designed by manipulating the SC stray fields. A large number of available spin textures may thus result from the chosen SC geometry and magnetic history.

Geometrical frustration has been the main strategy to generate nontrivial, monopolar‐like defects and other exotic states in magnetic materials such as spin ice systems or magnetic nanowires.^5^, ^6^, ^7^, ^8^, ^9^, ^10^, ^11^, ^12^, ^13^, ^14^ Skyrmions and other topological structures are typically created by artificially adding extra interactions, such as Dzyaloshinskii–Moriya interaction,^2^ to the conventional magnetic energy. However, these strategies often involve complex procedures and are sometimes restricted to confined regions of the phase diagram.^2^ It is thus essential to explore new approaches which incorporate versatility and freedom in encoding stable modifiable magnetic structures. Here we use the combination of superconductivity and magnetism in a hitherto unexplored way to imprint nontrivial spin textures. The interplay between these two antagonistic long‐range order phenomena, gives rise to rich physical properties and unusual behaviors.^15^, ^16^, ^17^ Their competing interactions in hybrid SC‐FM systems, have led to proximity effects,^15^, ^18^ stray‐magnetic field manipulation of superconductivity,^19^ detection of superconducting magnetic field distributions,^20^, ^21^ vortex guidance,^22^, ^23^ and spin‐injection phenomena,^17^, ^24^ among other effects. We have prepared a particular SC‐FM hybrid system consisting of high‐temperature YBa_2_Cu_3_O_7−δ_ superconducting dots, patterned with different shapes, and covered by a continuous thin ferromagnetic Permalloy (Py) layer, see **Figure** **1**a and Experimental Section. The in‐plane magnetic domain configurations of the Py layer are imaged by X‐ray photoelectron emission microscopy (X‐PEEM) using X‐ray magnetic circular dichroism (XMCD) as magnetic contrast mechanism.^25^, ^26^ X‐PEEM is an element selective technique capable to identify the magnetic moments of the Py layer, contrary to other magnetic imaging techniques such as Kerr, Lorentz or Magnetic Force Microscopy which are sensitive to the magnetic fields of both the SC and the Py layer.^27^ The magnetic contrast in the X‐PEEM images depicted here are measured at the Fe *L*_3_‐edge (707.6 eV) hence arising only from the Py magnetic domains. All measurements have been performed at magnetic remanence, after applying an out‐of‐plane external field up to a value of *B*_max_ (ranging between ± 30 mT). As indicated in the color scale of XMCD images, the magnetic contrast shown is proportional to the projection of the Py magnetization along the X‐ray beam direction (*x* axis as depicted in Figure 1b) being zero for magnetic moments oriented orthogonally to it.^26^

**Figure 1:**
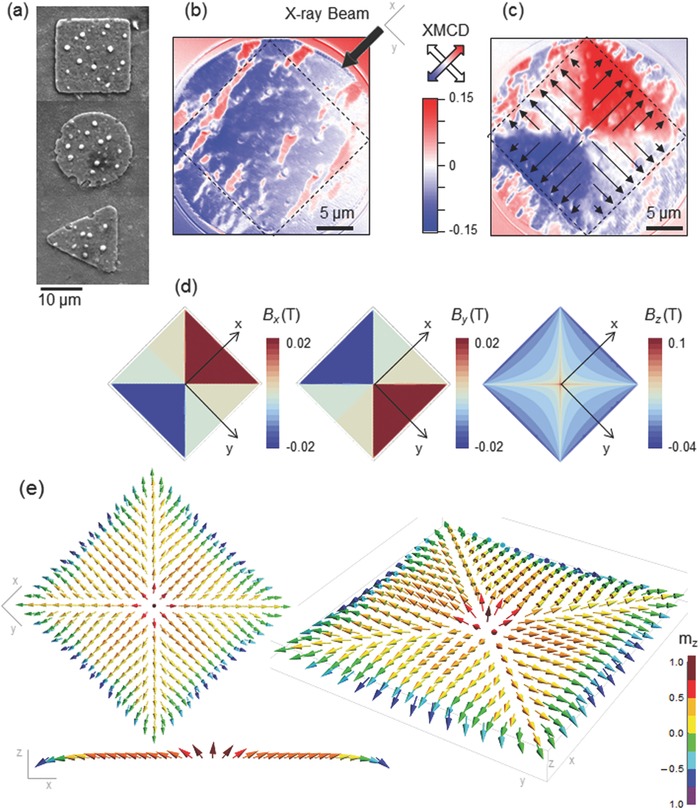
a) Scanning Electron Microscopy (SEM) image of SC‐FM hybrid structures with different SC dot geometry (square, disk, and triangle). b,c) XMCD images obtained at 45 K after zero field cooling (b) and at remanence after maximum applied field of +30 mT c) for a Py layer on top of a square SC dot. Dashed lines show the limits of the SC structure. The arrow in (b) shows the X‐ray beam direction and arrows in (c) indicate the direction of the magnetization in the Py layer. d) Simulation of the Cartesian components of the trapped field distribution in a saturated squared SC dot. e) 3D Py spin texture in the squared hybrid structure determined from micromagnetic simulations. Color scale shows the out‐of‐plane magnetization component.

Figure 1b shows the Py magnetic domain structure obtained on a 25 μm diameter area, including a 20 μm side squared SC dot, at *T* = 45 K, after cooling the hybrid structure at zero field. The image reveals an in‐plane stripe magnetic domain pattern, characteristic of a soft‐magnetic continuous thin film. No differences are apparent between Py regions with and without SC structure underneath, i.e., magnetic domains are not affected by the SC structure, evidencing that no interaction between the two layers is observed at this stage. However, a clear modulation of the Py magnetization emerges when the SC dot is in remanence after being magnetized to a maximum field *B*_max_ = +30 mT, at the same temperature (Figure 1c). XMCD images show that the Py magnetic moments on the squared structure adopt a singular magnetic configuration in which four tail‐to‐tail domain walls emerging from the square diagonals are formed and stabilized.

In general, the magnetization pattern of a ferromagnetic sample is determined by the interplay between the different competing contributions to the total energy. In the case of a Py film without external magnetic field these contributions are the magnetostatic and exchange energies. The magnetic anisotropy energy is not considered here because it can be neglected for a soft ferromagnetic material like Py. The magnetostatic energy is in general reduced by an in‐plane magnetization closing the magnetic flux, whereas the parallel alignment between neighboring spins would reduce the exchange contribution. The final spin distribution is a compromise between these two behaviors. According to this, the results of these interactions would lead to a Landau flux‐closure pattern, in the case of a micron sized permalloy square,^28^ or a 2D domain configuration (as that observed in Figure 1b) for a Py continuous film. In contrast, in our hybrid squared structure the spins adopt a highly unfavorable configuration in terms of magnetostatic energy in which tail‐to‐tail domains are induced.

To understand the formation of this singular spin texture in the FM layer, stable at zero external magnetic field, it is necessary to take into account the extra contribution to the system's total energy given by the remanent stray field generated by the SC dot, below the FM layer. The spatial distribution of supercurrents in a type‐II SC is highly dependent on its geometry. Here we take advantage of the SC thin film geometry, where supercurrents induced in response to the magnetic field that penetrates in the SC exhibit very large magnetization (and thus large stray fields) due to demagnetizing effects.^29^ For sufficiently high values of the applied field (as those used in the experiments) the path of the supercurrents simply follows the path of the external contour of the dot (circular, square or triangular in this work).^20^, ^29^ When the field applied to the SC is ramped up and down, regions with supercurrents circulating clockwise or counter‐clockwise are induced in alternating concentric layers,^29^ with a large variety of possible final stray magnetic field configurations. Figure 1d shows the Cartesian components of the trapped magnetic field distribution calculated for a squared SC dot in the saturated remanent state. By comparing the measured *x*‐component of the magnetization shown in Figure 1c with the trapped field distribution within the SC dot at this orientation, *B_x_* in Figure 1d, it is clearly observed that the trapped SC field pattern becomes basically imprinted into the Py layer. Micromagnetic simulations are used to calculate the 3D spin texture in the Py layer, considering the whole SC‐FM hybrid squared structure (Figure 1e).^30^ The obtained magnetic moment distribution confirms the in‐plane spin component measured experimentally. The stray field of the SC does not only stamp the four tail‐to‐tail domain pattern but also transforms the Néel domain walls typical of the Landau structure (the magnetization rotates toward the direction normal to the wall tangent) to Bloch domain walls (the magnetization rotates toward the domain‐wall tangent).^31^ The four domain walls merge at the center of the sample with out‐of‐plane spins induced by the *z*‐component of the SC stray field (*B_z_* in Figure 1d).

We next demonstrate how the magnetic history experienced by the SC‐FM hybrid controls the magnetic pattern imprinted in the FM. First panel of **Figure** **2**a (*B*_max_ = –30 mT) shows the four magnetic domains, with head‐to‐head spins, created in the negatively saturated state. Domains with opposite polarity appear at the edges of the squared hybrid structure, when the applied magnetic field direction is reversed (Figure 2a, *B*_max_ = +10 mT). Further increase of *B*_max_ leads to a controlled movement of the newly created domain walls toward the center of the structure. A complete reversal of the original domain pattern is obtained after saturating the SC dot with a positive magnetic field (Figure 2b, *B*_max_ = +30 mT). Domain walls with opposite polarity can then be induced by changing back the direction of the external field, recovering the initial magnetic pattern by saturating the structure with a negative field (Figure 2b, *B*_max_ = –25 mT). Figure 2c shows the *x*‐component of the Py spin texture (Cartesian magnetic component depicted in the XMCD images shown in Figure 2a,b, determined from micromagnetic calculations, considering a squared SC‐FM hybrid structure, for the different saturated SC states experimentally explored in Figure 2a. The good agreement between simulations and experimental data confirms the fact that the magnetic states induced in the FM layer are a faithful reproduction of the trapped fields in the SC.

**Figure 2:**
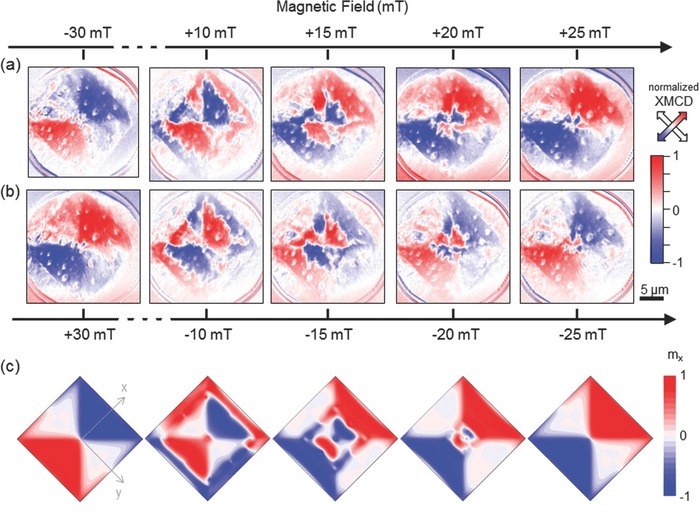
a,b) XMCD PEEM images of Py magnetic domains measured at *T* = 45 K and magnetic remanence, for a layer on top of a squared SC dot, after applying the different labeled maximum applied fields (*B*_max_). c) Micromagnetic simulation of the *x*‐component of the Py spin texture evolution, for a squared hybrid structure at the different remanent states shown in (a).

It is important to remark that the magnetic patterns are imprinted and controlled at very small fields (*B*_max_ ≤ ± 30 mT), and observed at zero applied field, thanks to the selected thin film geometry of the SC dots, which produce large demagnetizing effects giving large stray fields. Further outlook for applications would be the use of dots with smaller sizes that would be saturated at even lower fields. Regarding the temperature dependence of the phenomena, no significant differences may be expected in the spin pattern imprint by changing the temperature, as long as it is maintained low enough to ensure sufficient contribution of SC stray fields. By lowering the temperature larger SC magnetization would be obtained, thus enlarging the imprinting effect of the SC field pattern into the Py layer. In this case, however, larger applied fields would be required to saturate the dots.

To deeper understand the role of the SC on imprinting the FM spin textures in the Py layer we analyze the local magnetic response of different zones in the FM on top of a triangular SC structure (**Figure** **3**a). Space resolved magnetic X‐PEEM hysteresis loops (see Experimental Sections) extracted at different positions (Figure 3b) show lower coercivity *B_c_* values for Py regions near the edges of the SC triangular dot than in central positions. These differences arise from the fact that supercurrents penetrate in the SC dot from the surfaces inward and thus the external field, necessary to reverse the magnetic Py domains, reaches first the edge points than the inner ones. Figure 3c shows a scan line profile and Figure 3d a 2D map of the Py coercive field. *B_c_* increases as one approaches the center of the dot and drops to zero at the intersecting point of domains, where the stray field is basically cancelled. So, the coupling between the SC dot and the FM layer produces a spatial 2D Py coercive field modulation and domain wall manipulation that envisage these hybrid systems as a versatile nanoscale option to generate, modify, and annihilate a large number of singular spin configurations for potential applications in logic or memory devices.^32^, ^33^, ^34^

**Figure 3:**
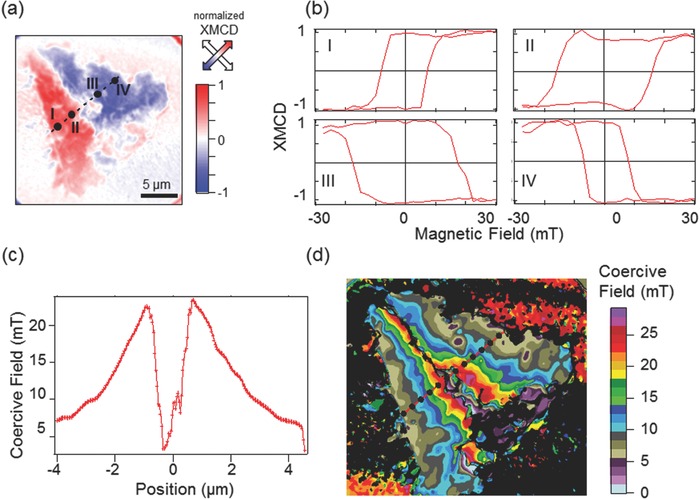
a) XMCD image of a triangular hybrid structure at remanence after a maximum applied field of –30 mT. b) Magnetic hysteresis loops of the Py layer at different positions over a triangular SC dot, labeled in (a). c) Scan line of the coercive field measured along the dashed line shown in (d). d) 2D map of the Py coercive field versus position.

The studied SC‐FM hybrid materials are ideal systems to encode magnetic topological defects. We show in **Figure** **4** the results for a SC disk structure in which the singular magnetic field distribution trapped in the SC stabilizes a 2D distribution of head‐to‐head or tail‐to‐tail magnetic moments pointing to or emerging from the center of the disk, depending on the sign of *B*_max_, at zero applied field. Line profiles of the XMCD signal versus angular position along a circumference within the disk (green line in Figure 4a,d), for the two magnetic states induced with *B*_max_ = ±30 mT are sinusoidal (Figure 4b,e). Since the XMCD signal is proportional to the projection of the magnetization along the beam propagation direction this shows that the magnetization direction of Py on top of the disk rotates around the structure with its center of rotation at the center of the SC circle. This magnetization profile corresponds to a 2D monopole‐like magnetic domain configuration, whose magnetic charge can be easily switched from +1 to –1 by changing the sign of *B*_max_. In the case of Py disks with sizes of submicron or few microns, the spin configuration with the lowest energy at zero applied magnetic field is the vortex‐state magnetization. This vortex state is characterized by an in‐plane curling magnetization (with a given chirality either clockwise or counter‐clockwise) and a nanometer size central vortex core region with an out‐of‐plane magnetization (with an up or down polarity). In our SC‐FM hybrid system, thanks to the remanent magnetic states created in the SC dot, a topological nontrivial monopole‐like spin texture is induced, in which all spins are pointing inward (outward) to the center of the disk.

**Figure 4:**
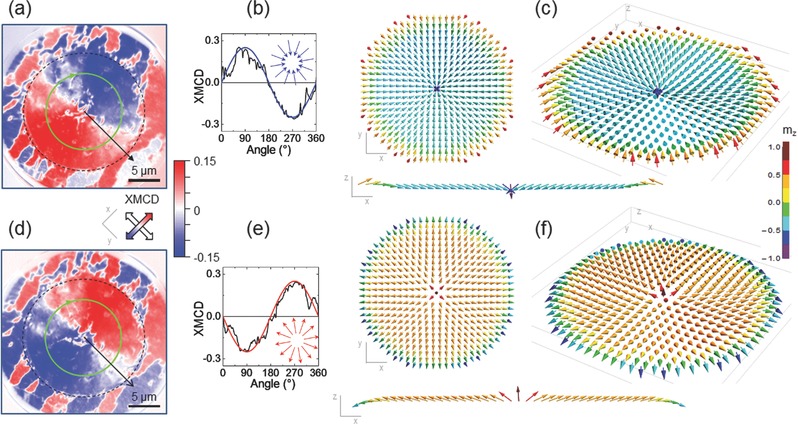
a) XMCD image of a disk hybrid structure at remanence after a maximum applied field of –30 mT. Dashed line shows the limits of the SC dot. b) XMCD signal along circumference (green solid line in (a)) versus angle. Solid curve shows the expected XMCD versus angle signal for a monopole field, depicted in the inset. c) Micromagnetic simulations showing the 3D spin texture of the Py layer in the disk hybrid structure. d–f) idem at +30 mT.

Micromagnetic simulations shown in Figure 4c,f reveal that the 3D orientation of the Py spins in the hybrid circular structure mainly follows the direction of the SC stray field, which is basically out‐of‐plane at the center of the sample and radially in‐plane (pointing inward or outward depending on the sense of the SC currents) away from the center. The effects of the magnetostatic and exchange interactions are limited to smoothing out the SC stray field direction. Thus, at the center of the sample there is a confined concentration of the magnetic poles with considerable out‐of‐plane oriented spins, with in‐plane components pointing inward or outward. The size of this central part, where the orientation of the spins is mainly out‐of‐plane, does not change with sample radius, *R*. The expansion of this region is avoided since the *z*‐component of the SC stray field increases as ln *R* whereas the magnetostatic field provided by the accumulation of the magnetic poles increases as *R*. Away from the center of the disk the *z*‐component of the field of the SC decreases and the in‐plane components totally control the spin behavior.

Notice that we have been able to entirely simulate the different encoded magnetic states in the SC‐FM hybrid systems by just considering the magnetostatic and exchange energies of the FM layer, in combination trapped fields within the SC dots. The influence of proximity effects at the SC‐FM interface thus may not be the determining mechanism behind the observed phenomena, giving simplicity in the modeling and design of exotic magnetic states on demand.

In conclusion, we have experimentally demonstrated and simulated an SC‐FM hybrid system that introduces a novel way to imprint, control, and erase stable nontrivial magnetic states in a ferromagnetic film, by using the stray fields of superconducting dots. The general procedure here presented (including experiments and simulations) can be easily expanded to provide a large number of magnetic topological defects of interest in a controlled way. The scheme introduced could be readily transferred to dots with diameters at the nanoscale. Possible extensions also include the use of elongated geometries (i.e., long rectangles instead of squares) for generating monopole defects extended along a line, and the possibility of feeding transport current in the superconductors.^29^ The use of nonsingle connected geometries in the SC dots (i.e., holes), where magnetic flux is quantized in the holes, and even nonconnected complex structures reveal new possibilities for imprinting novel interesting magnetic landscapes.

## Experimental Section

The hybrid structure was fabricated from a 250 nm superconducting YBa_2_Cu_3_O_7−δ_ (YBCO) film grown by chemical solution deposition on a 5 mm × 5 mm LaAlO_3_ single crystal substrate with a critical temperature of *T*_c_ = 92 K and self‐field critical current density *J*_c_^sf^(45 K) = 10 MA cm^–2^.^35^ Superconducting dots (20 μm × 20 μm) of different shapes were patterned using optical lithography and chemical wet etching. With these dimensions the dots could be considered as thin structures with thickness much smaller than the sample length. Patterned dots were in situ covered at the PEEM preparation chamber with a 4 nm Py, Fe_20_Ni_80_, soft ferromagnetic layer deposited by sputtering evaporation. It was checked that no degradation in the YBCO superconducting properties was obtained after the sputtering of Py layer. Figure 1a shows Scanning Electron Microscopy (SEM) images of SC‐FM hybrid structures with square, disk, and triangular SC dot geometry. White dots in the images are identified as superficial Cu containing particles, commonly observed in CSD YBCO films do not affect detrimentally the superconducting film properties.^36^ Detailed maps of the magnetic domain pattern within the Py layer were obtained by means of X‐PEEM at the UE‐49 PGMa beam‐line (Synchrotron BESSY II). For magnetic imaging the photon energy was tuned to the *L*_3_ resonance of iron (707.6 eV) exploiting the element‐specific XMCD. All X‐PEEM measurements were performed, with a field of view of 25 μm, in magnetic remanence, after applying an out‐of‐plane magnetic field (*B*_max_) to the hybrid structures previous to data acquisition, by a coil attached to the sample holder. Magnetic contrast images shown within the manuscript were obtained in two different ways: (i) XMCD images depicted in Figures 1 and 4 were calculated from a sequence of images taken with circular polarization (90% of circular photon polarization) and alternating helicity (σ^+^ and σ^−^, respectively). After normalization to a bright‐field image, the sequence was drift‐corrected, and frames recorded at the same photon energy and polarization were averaged. The magnetic contrast is shown as the difference of the two average images with opposite helicity, divided by their sum, i.e., (σ^+^ – σ^−^)/(σ^+^ +σ^−^), the so called XMCD asymmetry. (ii) Magnetic images depicted in Figures 2 and 3 were computed from element‐specific space‐resolved magnetic hysteresis loops. These magnetic loops were measured via a sequence of images taken at fixed helicity of the incoming circular polarization as a function of *B*_max_, increasing from –30 mT to +30 mT and back to –30 mT. As for the XMCD case all images were normalized to a bright‐field image and drift corrected. The image obtained from averaging all frames (*I*_aver_) contained no magnetic information within the SC region due to the symmetry of the in‐plane components of the magnetic field as a function of *B*_max_. This average image was then subtracted from the whole stack giving rise to the magnetic contrast images depicted in the figures. It was noted that the magnetic contrast obtained in such a way revealed only magnetic domains of Py on top of SC nanostructures. The magnetic domain pattern outside the SC regions does not change as function of *B*_max_ and is therefore equal to *I*_aver_ computed within these regions.

Micromagnetic simulations showing the spin arrangement in the Py layer were obtained by iteratively solving the Brown's equations.^30^ The Fe_20_Ni_80_ was simulated using an exchange stiffness *A* = 1.3 × 10^11^ J m^–1^, a saturation magnetization *M_S_* = 4 × 10^5^ A m^–1^, and magnetocrystalline anisotropy *K_U_* = 0. The sample was discretized in 10 nm × 10 nm × 4 nm cells (consistent with the exchange length). The magnetic field created by the currents in the SC dots was numerically calculated from the Biot–Savart law assuming the actual value of critical‐current density of the YBCO. The sizes of the simulated hybrid systems were 5 μm × 5 μm for the disk‐shaped sample and 2 μm × 2 μm for the squared sample, with a thickness of 4 and 250 nm for the Py and YBCO layers, respectively. Lateral dimensions smaller than the real samples were used (20 μm × 20 μm) because of the huge computing time, however the presented results can be extrapolated to larger structures.
